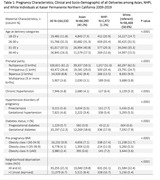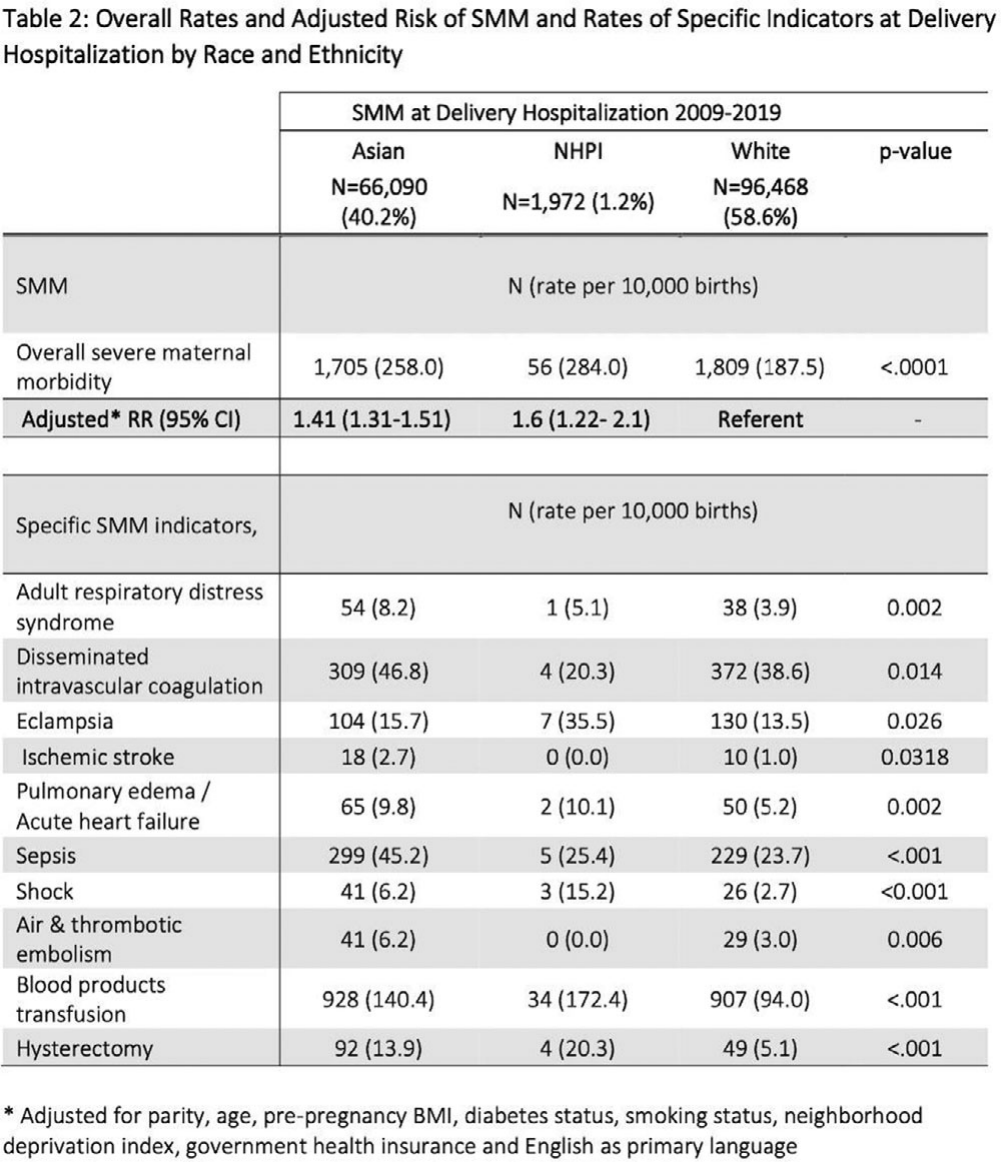# Oral Concurrent Session 1 – Equity, Public Health, and Policy: Thursday, January 30, 2025 1:30 PM – 4:00 PM

**DOI:** 10.1002/pmf2.12004

**Published:** 2025-01-05

## 9 | Dysregulated Gene Expression in Placentas with Exposure to Persistent Maternal Socioeconomic Disadvantage

Greg E. Miller^1^; Lauren Keenan‐Devlin^2^; Alexa A. Freedman^1^; Renee Odom‐Konja^3^; Linda M. Ernst^3^; Steve Cole^4^; Ann EB Borders^2^

*^1^Northwestern University, Chicago, IL; ^2^Endeavor Health, Evanston Hospital, Evanston, IL; ^3^Endeavor Health, Evanston, IL; ^4^University of California, Los Angeles, Los Angeles, CAMA*

1:30 PM ‐ 1:45 PM

**Objective**: Persistent socioeconomic disadvantage is associated with many adverse health outcomes across the lifespan. One hypothesis is these risks start during gestation from hardship‐related changes in placental gene expression. Studies in animal models have yielded results consistent with this hypothesis, but it has yet to be tested in large, diverse human cohorts.

**Study Design**: 605 patients with singleton pregnancies were recruited during their first trimester. Socioeconomic status (SES) data was collected using a standardized interview, and disadvantage was quantified by educational attainment and occupational prestige. Biopsies from distinct cotyledons of the placenta's chorionic villous layer were collected < 2 hours from delivery and used for genome‐wide expression profiling via RNA‐Seq on an Illumina NovaSeq X Plus. Data were analyzed with linear mixed models, adjusted for maternal age, race, ethnicity, BMI, obstetric risk, delivery mode, and fetal sex.

**Results**: 449 participants delivered at term and had complete data. At a threshold of 1.50 fold‐change, analyses identified 1,859 transcripts that were differentially expressed across the 4 standard deviation range of SES. 1,311 transcripts were upregulated in conjunction with SES disadvantage and 548 were downregulated. Bioinformatic analyses yielded indications that SES disadvantage was associated with upregulated pro‐inflammatory transcriptional pathways (those anchored by GATA, STAT, MAF, and IRF) in fetal myeloid cells, but also with downregulation of trophoblast transcriptional pathways involved in fetal organ maturation (those anchored by PBX, c‐ETS, ELK1, PAX, and CREB).

**Conclusion**: These results suggest molecular correlates of maternal disadvantage during pregnancy are present in mRNA from the placenta chorionic villous layer demonstrating SES disparities in placental biology. Multiple dimensions of placental gene expression were dysregulated with exposure to SES disadvantage. Future work should focus on interventions that reduce SES disadvantage before and during pregnancy, and evaluate their impact on pregnancy outcomes and placental biology.

## 10 | Association Between Maternal‐Fetal Medicine Physician Density and Adverse Pregnancy Outcomes

Tetsuya Kawakita; Rula Atwani; Lindsay S. Robbins; George R. Saade

*Macon & Joan Brock Virginia Health Sciences Eastern Virginia Medical School at Old Dominion University, Norfolk, VA*

1:45 PM ‐ 2:00 PM

**Objective**: To examine the association between maternal‐fetal medicine (MFM) physician density and adverse pregnancy outcomes at the state level. 

**Study Design**: This was a cross‐sectional analysis of publicly available state‐level birth certificate data from 2018 to 2021. The number of MFM physicians per state each year was obtained from the American Medical Association database. The primary exposure was based on the density of MFM per state categorized by quartiles of the ratio of MFM in each state per 100,000 live births (low, fair, moderate, and high). Our primary outcome was maternal mortality rate during pregnancy and up to 42 days postpartum (MMR). Our secondary outcomes were maternal mortality rate up to 365 days postpartum, stillbirth rate, preterm birth rate, and cesarean delivery rate. We calculated incident rate ratios (IRR) with 95% confidence intervals (95%CI) using generalized estimating equations with Poisson distribution and exchange‐correlation structure, accounting for confounders.

**Results**: The median state MFM density was 31.6 MFM per 100,000 births (interquartile 21.9‐42.5). Of 14,803,520 births included in this analysis, 1,683,691 (11.4%) were in low MFM‐density states, 5,193,402 (35.1%) births were in fair MFM‐density states, 4,557,005 (30.8%) were in moderate MFM‐density states, and 3,369,422 (22.8%) were in the high MFM‐density states. There was an inverse relationship between the MFM density in the state and its MMR per 100,000 live births (Figure 1). Compared to the low MFM‐density states, high MFM‐density states were associated with a lower risk of MMR, PRM, and preterm birth (Table 1). There were no significant differences in outcomes between low MFM‐density states and fair or moderate MFM‐density states except for stillbirths being lower in the moderate MFM‐density states compared to the low MFM‐density states.

**Conclusion**: High MFM‐density states have a decreased risk of maternal mortality compared to low MFM‐density states, suggesting an association between MFM physician availability and decreased maternal mortality.

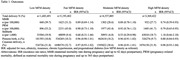

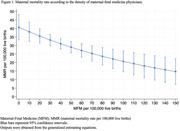

## 11 | Black Maternal Morbidity and its Association with Systemic Racism

Sebastian Z. Ramos; Meghan I. Short; Erika F. Werner; Chloe E. Bird; Ndidiamaka Amutah‐Onukagha; Michael B. Siegel

*Tufts University School of Medicine, Boston, MAMA*

2:00 PM ‐ 2:15 PM

**Objective**: To explore the relationship between systemic racism and Black maternal morbidity using the Systemic Racism Index (SRI), a county‐level measure that captures disparities in incarceration, education, economic status, employment, and segregation in Black versus White populations in the United States.

**Study Design**: Using CDC Vital Statistics data from 2019‐2021, a Composite Adverse Maternal Outcome Score (CAMOS)—which included blood transfusion, ruptured uterus, unplanned hysterectomy, and intensive care unit admission—was calculated for Non‐Hispanic Black (NHB) and Non‐Hispanic White (NHW) patients. The Systemic Racism Index and its relationship to rates of morbidity in NHB and NHW patients were studied. Mixed‐effects logistic regression models were fit, adjusting for individual level clinical factors and county‐level community vulnerability using the CDC's Social Vulnerability Index (SVI).

**Results**: There were 5,703,315 births for which covariates were measured in the 1,181 counties with an SRI score. The CAMOS rate in NHB patients was 7.6 vs 4.9 per 1000 in the NHW population. After adjusting for overall community vulnerability using the Social Vulnerability Index and individual level clinical and demographic factors, there was a 20% increase in CAMOS rate in NHB patients for each standard deviation increase in the SRI factor score (aOR 1.20, 95% CI 1.14, 1.26) and an 11% increase for NHW patients (aOR 1.11, 95% CI 1.06‐1.15).

**Conclusion**: There is a significant relationship between systemic racism, as measured by the Systemic Racism Index, and maternal morbidity disparity in NHB and NHW patients. Even after controlling for individual risk factors and overall community vulnerability, there was a strong correlation between the Systemic Racism Index and maternal morbidity, suggesting a mechanism separate from resource allocation and access to a more deeply entrenched system that perpetuates inequity. These findings underscore the profound impact of systemic racism on maternal health outcomes and highlight the need for addressing structural inequalities to reduce racial disparities in maternal morbidity.

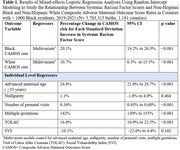

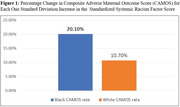

## 12 | Low‐income Birthing Individuals’ Need for and Satisfaction with Services in a One‐Year Postpartum Navigation Program

Maya J. Daiter^1^; Laura Diaz^1^; Hannah M. Green^1^; Ying Cheung^1^; Viridiana Carmona‐Barrera^1^; Brittney R. Williams^1^; Charlotte M. Niznik^1^; Joe M. Feinglass^1^; William A. Grobman^2^; Lynn M. Yee^1^

*^1^Northwestern University Feinberg School of Medicine, Chicago, IL; ^2^The Ohio State University, Columbus, OH*

2:15 PM ‐ 2:30 PM

**Objective**: Patient navigation seeks to reduce barriers to care and to overcome adverse social determinants of health. Navigating New Motherhood (NNM) is a trial in which low‐income individuals are randomized to receive postpartum navigation for one year. We aimed to understand what services were most needed by participants along with satisfaction with the navigation program.

**Study Design**: This is a mixed methods analysis of data from NNM participants randomized to navigation. All navigated participants completed study visits at 4‐12‐weeks (“12 weeks”) and 11‐13 months (“1 year”) postpartum. Visits included the 26‐item “Patient Satisfaction with Navigation‐Logistical (PSN‐L)” measure, which queried about services needed and‐for those services that were needed–satisfaction with the services received; the PSN‐L is scored from ‐3.31 to +3.43 (higher scores indicate greater satisfaction). A randomly‐selected sample of navigated participants also underwent in‐depth qualitative interviews at 4‐6 and 11‐13 months postpartum. Transcripts from both interviews, which were focused on perception and utilization of navigation services, were analyzed via the constant comparative method. Themes were identified using a concurrent triangulation design using survey and interview data.

**Results**: Of 203 navigated participants, 201 completed the PSN‐L at 12 weeks and 189 at 1 year. On nearly every PSN‐L item at both time points, >50% of participants indicated need for navigation service, with similar types of needs at both time points (Fig 1). The mean PSN‐L scores were 3.13 (SD 0.62) at 12‐weeks and 3.14 (SD 0.59) at 1‐year postpartum, revealing high satisfaction overall. Similarly, more than 90% of participants reported on the PSN‐L that they were “very satisfied” with most domains (20/26 at 12 weeks and 23/26 at 1 year) of navigation services provided. These findings were corroborated by thematic analysis (Fig 2).

**Conclusion**: While most participants expressed that they needed many services to optimize their health, they reported they were very satisfied with the capacity of patient navigation to address those needs.

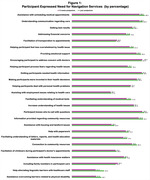

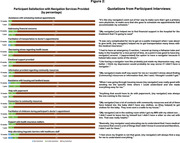

## 13 | Impact of WIC Enrollment on Adverse Maternal and Neonatal Adverse Outcomes in Medicaid Populations

Reetam Ganguli^1^; Stephen Wagner^2^

*^1^Elythea, San Jose, CA; ^2^Beth Israel Deaconess Medical Center, Boston, MA*

2:30 PM ‐ 2:45 PM

**Objective**: To evaluate the impact of the Women, Infants, and Children (WIC) program enrollment on severe maternal morbidities and neonatal adverse outcomes among pregnant Medicaid recipients from 2018 to 2022.

**Study Design**: A retrospective cohort study was conducted using data from the CDC National Vital Statistics System to compare maternal and neonatal outcomes between Medicaid‐insured pregnant women enrolled in WIC (n = 4,552,866) and those not enrolled in WIC (n = 2,948,168). Multivariate logistic regression was used to calculate adjusted odds ratios (aORs) with 95% confidence intervals (CIs) for various outcomes, adjusting for age, ethnicity, marital status, education status, and birth order.

**Results**: Enrollment in WIC was associated with a statistically significant reduction in several adverse outcomes. Women enrolled in WIC had a lower risk of postpartum hemorrhage (aOR 0.93, 95% CI [0.91‐0.95], p < 0.001), unplanned hysterectomy (aOR 0.98, 95% CI [0.92‐1.05], p = 0.014), and admission to the ICU (aOR 0.86, 95% CI [0.84‐0.89], p < 0.001). Neonatal outcomes also improved among WIC participants, with reduced odds of NICU admission (aOR 0.90, 95% CI [0.90‐0.91], p < 0.001) and low birth weight (aOR 0.91, 95% CI [0.91‐0.92], p < 0.001). Furthermore, WIC enrollment was associated with higher rates of breastfeeding at discharge (aOR 0.84, 95% CI [0.84‐0.84], p < 0.001).

**Conclusion**: Medicaid‐insured pregnant women enrolled in the WIC program demonstrated a significantly lower risk of adverse maternal and neonatal outcomes compared to those not enrolled in WIC. Accessible and innovative outreach and engagement strategies to improve WIC enrollment among pregnant Medicaid members may be vital in reducing preventable preterm labor, NICU admissions, low birth weight infants, and severe maternal morbidities among health insurance plans.

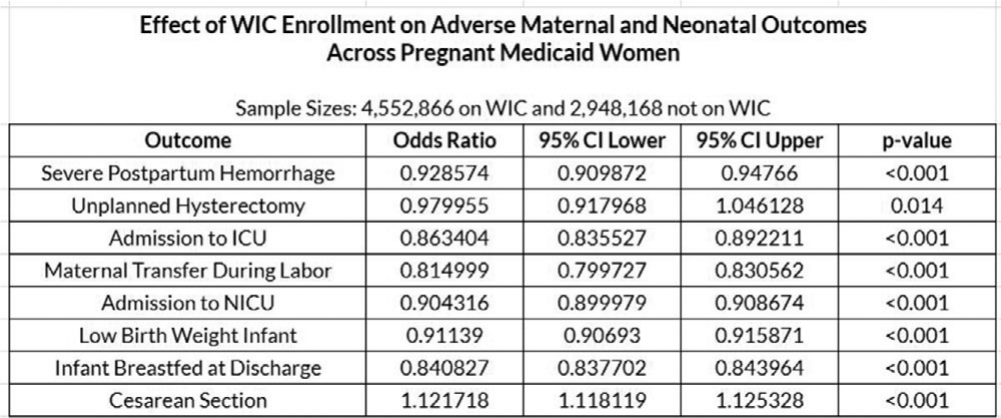

## 14 | Homicide and Suicide: The Leading Cause of Maternal Death, and How Firearm Legislation Affects It

Hooman A. Azad^1^; Dana Goin^2^; Lisa Nathan^3^; Dena Goffman^1^; Uma M. Reddy^4^; Danielle Laraque‐Arena^2^; Mary E. D'Alton^1^

*^1^Columbia University Medical Center, New York, NY; ^2^Columbia University Mailman School of Public Health, New York, NY; ^3^Columbia University Irving Medical Center, New York, NY; ^4^Columbia University, New York, NY*

2:45 PM ‐ 3:00 PM

**Objective**: In medical training, the dogma is this: the leading causes of maternal mortality are bleeding, infection, hypertension, and cardiovascular disease. This is only true when violence against women is excluded. We present the leading causes of death in pregnancy, compare violent death rates in pregnant and non‐pregnant people, and investigate firearm legislation's association with these deaths.

**Study Design**: Case‐level data on deaths in US women ages 15‐44 (2005‐2022) were collected using CDC's Limited Mortality File. Cause of death and pregnancy status were identified by *ICD‐10* codes. A “pregnancy checkbox” identified additional deaths in pregnancy. We include the first 42 postpartum days in defining “pregnancy,” consistent with national definitions.

We present the leading causes of death in pregnancy. We compare violent death rates among pregnant women and non‐pregnant controls. A state‐level, cross‐sectional, ecologic panel study using negative binomial regressions is used to identify associations between domestic violence (DV) firearm laws and violent deaths in pregnancy.

**Results**: In 18 years, 20,421 pregnant women died. 2,293 were violent deaths (11%); of these, 1,407 (61%) were homicides and 886 (39%) were suicides. 1,261 violent deaths involved firearms (55%). Violence was the most common cause of death in pregnancy (Figure 1A); the incidence doubled from 2005‐2022. Violent death was more frequent in pregnant women than non‐pregnant controls (3.2 v 1.2 deaths per 100,000 population) (Figure 1B).

DV laws were associated with reductions in homicide and firearm death in pregnancy (range, 17‐32%). Laws requiring relinquishment of firearms among DV perpetrators showed the strongest associations with reduced death rates (Table 1).

**Conclusion**: More pregnant women die from violence than any medical cause in the US, at higher rates than their non‐pregnant counterparts. Firearm legislation is associated with fewer deaths, suggesting lethal means restriction may help curb this alarming trend. As maternal mortality rates rise, addressing violence should be a focus of clinicians and policymakers alike.

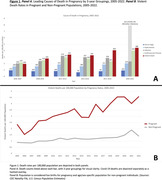

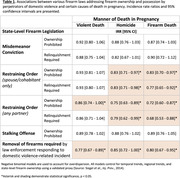

## 15 | Elimination of a Threefold Racial Disparity in Aspirin Recommendation Using Integrated Clinical Decision Support

Melissa S. Wong^1^; Rommy Coutelin Johnson^2^; Karla Gonzalez^1^; Ojiugo Onwumere^3^; Kristin Parrinella^4^; Camelita Thrift^3^; Samira Torna^1^; Matthew Wells^1^; Kimberly D. Gregory^3^

*^1^Cedars‐Sinai Medical Center, Los Angeles, CA; ^2^Cedars‐Sinai, Los Angeles, CA; ^3^Cedars Sinai Medical Center, Los Angeles, CA; ^4^Kaiser‐Permanente, Los Angeles, CA*

3:00 PM ‐ 3:15 PM

**Objective**: Low dose aspirin (LDA) for preeclampsia prevention is under‐prescribed and Black pregnant people are disproportionately affected. Clinical decision support (CDS) embedded in the electronic health record (EHR) has been shown to improve compliance. The aim of this study was to evaluate whether the presence of a CDS tool (1) increased recommendation of LDA and (2) reduced the racial disparity.

**Study Design**: We performed a pre/post implementation study comparing two time periods: Oct 2019‐Mar 2020 and Oct 2023‐Mar 2024; parallel months were chosen to limit seasonal changes. We used manual chart review to assess patient factors (Table 1) and to identify LDA recommendation. Our primary outcome was the rate of LDA recommendation (documented discussion or order placed). We assessed rates of hypertensive disorders of pregnancy, obstetric and neonatal outcomes, and performed subgroup analysis for Black patients to determine whether there were differences in LDA recommendation.

**Results**: There were 677 patients in the pre‐CDS and 649 in the post‐CDS period; similar proportions met criteria to receive LDA (Table). A significantly greater proportion received LDA after the implementation of the CDS (pre‐23.5%; post‐78.1% p = < .0001) (Figure).

In the pre‐CDS period, Black race was associated with even fewer recommendations (Black [10.4%] vs. non‐Black [26.5%] p = 0.02). After implementation of the CDS, the racial disparity was eliminated (Black [82.1%] vs. non‐Black [77.6%]), p = 0.54).

There were no differences in the rates of hypertensive disorders of pregnancy, obstetric or neonatal outcomes.

Despite CDS triggering for only 109 (40%) of those who met criteria, the LDA use increased for all meeting criteria (even those for whom the CDS did not trigger, e.g., a patient already on LDA).

**Conclusion**: The use of CDS for LDA prophylaxis tripled LDA use, eliminated a threefold racial disparity, did not increase adverse outcomes, and increased LDA use even for those for whom the CDS did not trigger. This suggests physician learning and behavior change contributed to increased compliance in addition to the CDS.

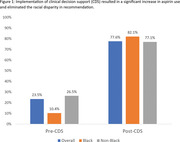

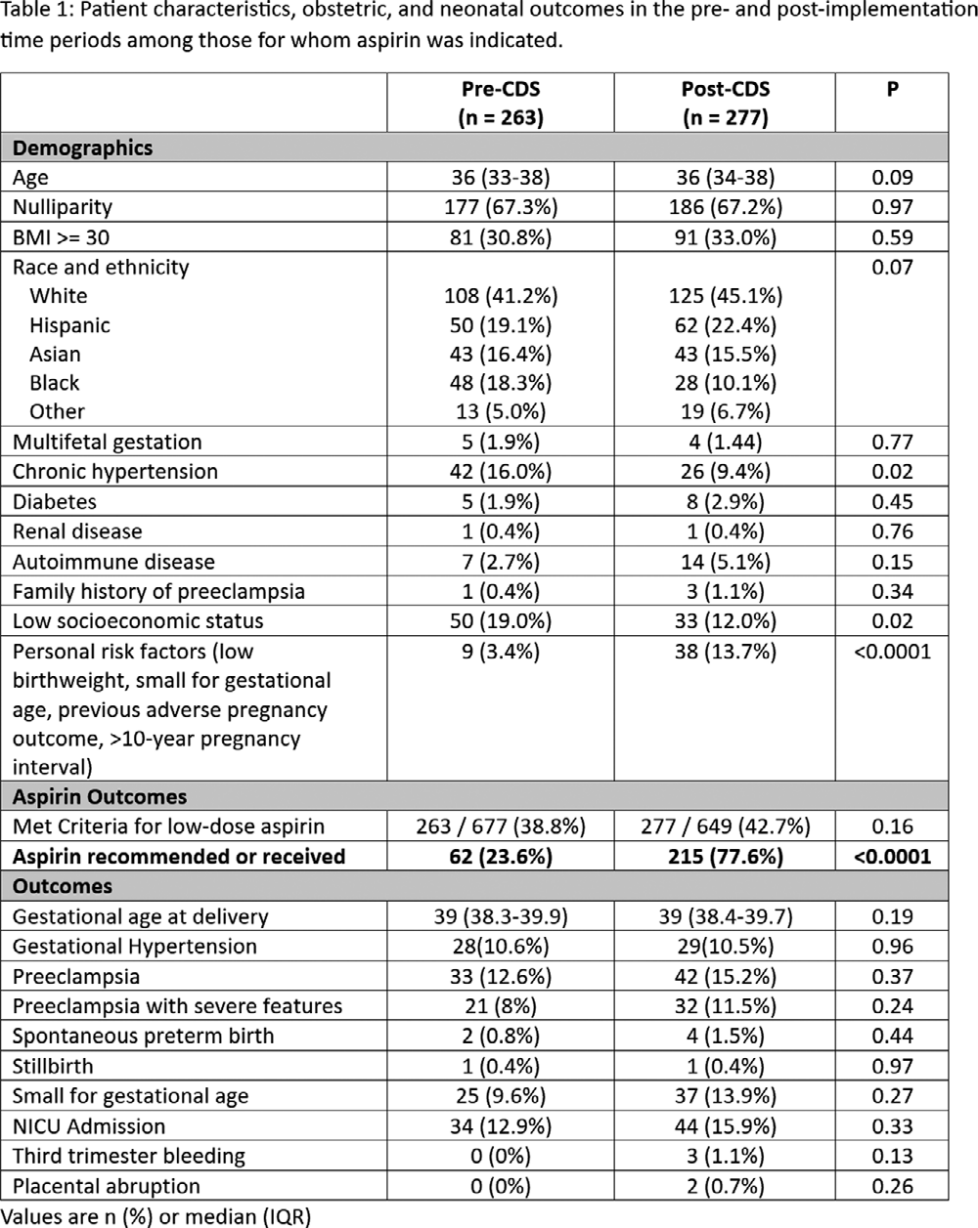

## 16 | Severe Maternal Morbidity Differences by State Medicaid Expansion and Abortion Coverage: National Database Analysis, 2014‐2022

Ishana Shetty; Mark C. Valentine; Mark Hoofnagle; Jennifer Reeves; David L. Eisenberg

*Washington University, St. Louis, MO*

3:15 PM ‐ 3:30 PM

**Objective**: The objective of this study was to compare the rate of severe maternal morbidity (SMM) state by state stratified by whether that state's Medicaid 1) underwent expansion under the Affordable Care Act (ACA) and 2) had coverage of abortion care beyond Hyde requirements.

**Study Design**: This was a retrospective cohort study using data on state level SMM rates (reported per 10,000 in‐hospital deliveries) from 2014–2022 using the publicly available Healthcare Cost and Utilization Project (HCUP) Fast Stats dataset. We used a negative binomial generalized linear model to analyze SMM as a function of year and state with separate analysis by Medicaid expansion under the ACA and state Medicaid coverage of abortion care beyond Hyde requirements. Categorization of states was based on Kaiser Family Foundation designation.

**Results**: The rate of SMM was significantly reduced in states expanding Medicaid (0.907, 95% CI 0.852‐0.967). Among the states that expanded Medicaid, the rate of SMM was further reduced in states that also expanded abortion coverage (0.920, 95% CI 0.856‐0.988). When SMM was modeled as a function of state, year, and Medicaid/abortion expansion (i.e. neither, one, or both), we saw a decrease in SMM for expanding either Medicaid or abortion (0.985, 95% CI 0.829‐1.16) and a larger decrease for expanding both (0.936, 95% CI 0.844‐1.038), though neither trend reached significance.  

**Conclusion**: The rate of SMM was significantly reduced in states that expanded Medicaid under the ACA compared to states that did not. Medicaid expanded states that additionally provided abortion coverage under Medicaid saw further reductions in the rate of SMM. When modeled simultaneously, Medicaid expansion and abortion expansion both trend towards decreased SMM, with expansion of both showing the largest decrease, although this did not reach significance in this data set. These findings add to growing literature that national policies like abortion restriction and Medicaid expansion significantly impact maternal health outcomes.

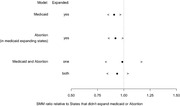

## 17 | Elevated Micro‐ and Nanoplastics Detected in Preterm Human Placentae

Enrico R. Barrozo^1^; Marcus A. Garcia^2^; Michael D. Jochum, Jr.^1^; Rui Liu^2^; Alex Nihart^2^; Eliseo Castillo^2^; Eliane El Hayek^2^; Jorge Gonzalez‐Estrella^3^; Lori Showalter^1^; Cynthia Shope^1^; Melissa A. Suter^1^; Matthew J. Campen^2^; Kjersti M. Aagaard^4^

*^1^Baylor College of Medicine and Texas Children's Hospital, Houston, TX; ^2^University of New Mexico, Albuquerque, NM; ^3^Oklahoma State University, Stillwater, OK; ^4^Boston Children's Hospital, Division of Fetal Medicine and Surgery, Boston, MA; HCA Healthcare and HCA Healthcare Research Institute, Nashville, TN; HCA Texas Maternal Fetal Medicine, Houston, TX; Baylor College of Medicine and Texas Children's Hospital, Houston, TX, Boston, MA*

3:30 PM ‐ 3:45 PM

**Objective**: We and others recently demonstrated the widespread presence of micro‐ and nanoplastics (MNPs) in the environment and their documented absorption by humans, with deposition and bioaccumulation in the placenta. In the current study, we aimed to test the hypothesis that placental MNP concentrations and species differ between term and preterm birth.

**Study Design**: We analyzed human placentae collected at term (n = 100) and preterm (< 37 weeks gestation; n = 75). Placental specimens were subjected to pyrolysis‐gas chromatography/mass spectrometry (Py‐GC/MS) to quantify twelve types of MNPs: polyethylene (PE), polypropylene (PP), acrylonitrile butadiene styrene (ABS), styrene‐butadiene rubber (SBR), polymethyl methacrylate (PMMA), polycarbonate (PC), polyvinyl chloride (PVC), polyurethane (PU), polyethylene terephthalate (PET), nylon 6 (N6), and nylon 66 (N66). For rigor & reproducibility, duplicate specimens were run & quantified relative to standards.

**Results**: Highly sensitive Py‐GC/MS demonstrated significantly higher levels of PC, PVC & N66 MNPs in preterm placentae compared to term (Fig 1A; q = 0.002, 0.006, 0.013, respectively, by mixed‐effects model with Sidak's multiple corrections). Moreover, despite shorter gestations, cumulative MNP concentrations were higher in preterm placentae (Fig 1B; preterm mean 203.0 µg/g tissue ± 19.41 SEM vs term mean 129.8 µg/g tissue ± 14.16 SEM, p< 0.0001 Mann‐Whitney). Stratified analyses showed significant differences by fetal sex (Fig 1C) and race/ethnicity (Fig 2A) (q< 0.05), and a significant correlation between social deprivation indices (SDI) and MNP levels (Fig 2B). Total MNP levels in placentae were 121.9 times higher than those reported in human blood (p< 0.0001 Mann‐Whitney).

**Conclusion**: Our findings suggest that MNPs accumulate in placentae from preterm births. Given the established link between MNPs and inflammation, we speculate that MNP bioaccumulation may play a role in modulating inflammatory preterm birth. These novel findings underscore the importance of prioritizing mechanistic studies to explore how MNPs impact fetal development and pregnancy outcomes.

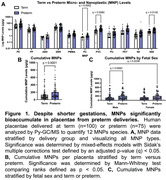

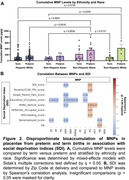

## 18 | Severe Maternal Morbidity among Asian and Pacific Islander Parturients in a Contemporary Northern California Cohort

Shalila A. de Bourmont^1^; Janet Alexander^2^; Baiyang Sun^2^; Erica P. Gunderson^2^; Mara Greenberg^1^

*^1^Kaiser‐Permanente Northern California, Oakland, CA; ^2^Kaiser‐Permanente Northern California, Pleasanton, CA*

3:45 PM ‐ 4:00 PM

**Objective**: To compare rates and types of severe maternal morbidity (SMM) among Asian, Native Hawaiian/Pacific Islander (NHPI), and White pregnant individuals in a large integrated health care delivery system.

**Study Design**: Retrospective cohort study of singleton gestations delivered ≥ 20 weeks in Kaiser Permanente Northern California (KPNC) 2009‐2019, self‐identified as Asian, NHPI or White. SMM was defined as presence at least 1 of 21 indicators per CDC criteria. Maternal characteristics at time of entry to prenatal care and obstetric comorbidities were compared between groups with White as the referent group. Absolute rates (cases per 10,000 births) of SMM and each SMM indicator at delivery hospitalization were compared between groups using chi‐square statistics and logistic regression to estimate the adjusted relative risks for Asian and NHPI relative to the White subgroup.

**Results**: Among the 164,530 singleton deliveries analyzed, groups significantly differed in all maternal characteristics and obstetric comorbidities. NHPI patients were more likely to have higher rates of chronic and gestational hypertension, preeclampsia and pregestational diabetes (Table 1). SMM rates at delivery were significantly higher for NHPI (284/10,000) and Asian (258/10,000) subgroups than for the White referent group (p< 0.001,Table 2). Rates of SMM indicators differed between groups with higher rates of eclampsia, pulmonary edema/heart failure, shock, hysterectomy and blood products transfusion among NHPI; and higher rates of sepsis and DIC among Asian patients compared to White patients (all indicators p< 0.005, Table 2). Adjusting for covariates, the risk of SMM was higher for NHPI (RR 1.6; 95% CI 1.22‐2.1) and Asian (RR 1.41; 95% CI 1.31‐1.51) groups compared to the White referent group.

**Conclusion**: In this large cohort of NHPI, Asian and White patients, risk of SMM was highest for NHPI. Differences in rates of absolute SMM and SMM indicators were significant between groups highlighting the importance of disaggregating them in research and clinical settings to tailor care and reduce the disproportionately high rates of SMM.